# Fast Identification of Radical Scavengers from *Securigera varia* by Combining ^13^C-NMR-Based Dereplication to Bioactivity-Guided Fractionation

**DOI:** 10.3390/molecules200814970

**Published:** 2015-08-14

**Authors:** Pacôme Sientzoff, Jane Hubert, Coralie Janin, Laurence Voutquenne-Nazabadioko, Jean-Hugues Renault, Jean-Marc Nuzillard, Dominique Harakat, Abdulmagid Alabdul Magid

**Affiliations:** ICMR, UMR CNRS 7312, Campus Moulin de la Housse BP 1039, Reims 51687, France; E-Mails: Pacome.sientzoff@etudiant.univ-reims.fr (P.S.); coralie.janin@etudiant.univ-reims.fr (C.J.); laurence.voutquenne@univ-reims.fr (L.V.-N.); jh.renault@univ-reims.fr (J.-H.R.); jm.nuzillard@univ-reims.fr (J.-M.N.); dominique.harakat@univ-reims.fr (D.H.); abdulmagid.alabdulmagid@univ-reims.fr (A.A.M.)

**Keywords:** *S. varia*, Fabaceae, DPPH, flavonoids, centrifugal partition extraction, dereplication, ^13^C-NMR

## Abstract

*Securigera varia* (Fabaceae) is a common herbaceous perennial plant widely growing in Europe and Asia and purposely established for erosion control, roadside planting, and soil rehabilitation. The aim of this study was to determine the radical scavenging activity of a crude methanol extract of *S. varia* aerial parts by using the free radical DPPH (1,1-diphenyl-2-picrylhydrazyl) and to rapidly identify the compounds involved in this activity. The crude extract was initially separated in five fractions on Diaion HP20 resin and the most active part was fractionated by Centrifugal Partition Extraction (CPE). Known compounds were directly identified by a ^13^C-NMR-based dereplication method. Semi-preparative high performance liquid chromatography purification experiments were further performed to identify unknown or minor active compounds. As a result, one new (**13**) and twelve known flavonoid glycosides together with three nitropropanoylglucopyranoses were isolated, including astragalin (**1**), kaempferol-3-*O*-(6-*O*-acetyl)-β-d-glucopyranoside (**2**), kaempferol-3,4′-di-*O*-β-d-glucopyranoside (**3**), trifolin (**4**), isoquercitrin (**5**), hyperoside (**6**), isovitexin (**7**), isoorientin (**8**), isovitexin 4′-*O*-β-d-glucopyranoside (**9**), apigenin 7-*O*-β-d-glucuronopyranoside (**10**), luteolin 7-*O*-β-d-glucuronopyranoside (**11**), apigenin 7-*O*-α-l-rhamnopyranosyl-(1→2)-β-d-glucuronopyranoside (**12**), apigenin 7-*O*-β-d-glucopyranosyl-(1→2)-β-d-glucuronopyranoside (**13**), 6-*O*-(3-nitropropanoyl)-β-d-glucopyranoside (**14**), coronillin (**16**) and coronarian (**15**). 120 mg of the most active compound isoorientin against the free radical DPPH was recovered by CPE with an HPLC purity of 99%.

## 1. Introduction

In recent years, one area which attracted a great deal of attention is the possible therapeutic use of antioxidants to control degenerative diseases associated with marked oxidative damage [1,2]. This led to an increasing interest in natural substances with antioxidant properties [3]. Phenolic substances are recognized as natural antioxidants and have received much attention due to their role in the neutralization or scavenging of free radicals [4]. Flavonoids are classified as mixed antioxidants [5] because they are able to donate protons to free radicals, and are still able to prevent the formation of reactive oxygen species (ROS) either through inhibition of enzymes involved in oxidative processes, or by chelating metal traces involved in their production [6]. The role of flavonoids as antioxidants has also attracted interest due to their pharmacological properties related to protection against cardiovascular diseases [4].

*Securigera varia* (synonym *Coronilla varia*) is a creeping, perennial legume belonging to the Fabaceae family [7]. Its chemical composition includes nitropropanoylglucopyranoses, cardenolides, and flavonoids [7,8,9,10]. *S. varia* is cardiotonic [11] and the seeds of this plant have shown a potential antitumour activity due to their cardenolide content [12,13], whereas the MeOH extract of its aerial parts have demonstrated an antibacterial activity [14]. However, the antioxidant activity of this plant has never been reported. The aim of this study was to evaluate the radical scavenging activity of a crude methanol extract of *S. varia* aerial parts by using the stable free radical DPPH (1,1-diphenyl-2-picrylhydrazyl) and to rapidly identify the compounds responsible for the observed activity. For this purpose, the crude extract was initially separated in five fractions on Diaion HP20 resin and the most active one was fractionated by Centrifugal Partition Extraction (CPE). The known compounds recovered as simplified mixtures in the CPE-generated fractions were directly identified by a recently developed dereplication method [15] and tested against the stable radical DPPH. This method consists in ^13^C-NMR analyses of the fraction series, automatic collection and binning of ^13^C signals across spectra and hierarchical clustering analysis (HCA) of the resulting dataset. The aim of this pattern recognition approach is to measure the statistical correlations between ^13^C-NMR signals in the spectra of successive fractions in order to determine the presence of individual compounds by visualizing their carbon skeletons as “chemical shift clusters”. These clusters are assigned to molecular structures by using an in-house ^13^C-NMR database of natural metabolites containing the predicted ^13^C chemical shifts of all known *S. varia* metabolites [7,8,9,10,11,12,13]. This dereplication approach was completed by further semi-preparative high performance liquid chromatography purification experiments in order to identify unknown or minor active compounds. As a result, one new (**13**) and twelve known flavonoid glycosides (**1**–**12**) together with three nitropropanoylglucopyranoses (**14**–**16**) were isolated from the aerial parts of *Securigera varia*. The structures were established by NMR and mass spectrometry as well as by comparison to their spectral data (MS, ^1^H- and ^13^C-NMR) found in the literature. Structure-activity relationships between the identified molecular structures were identified and their free radical scavenging activity were also investigated.

## 2. Results and Discussion

**Figure 1:**
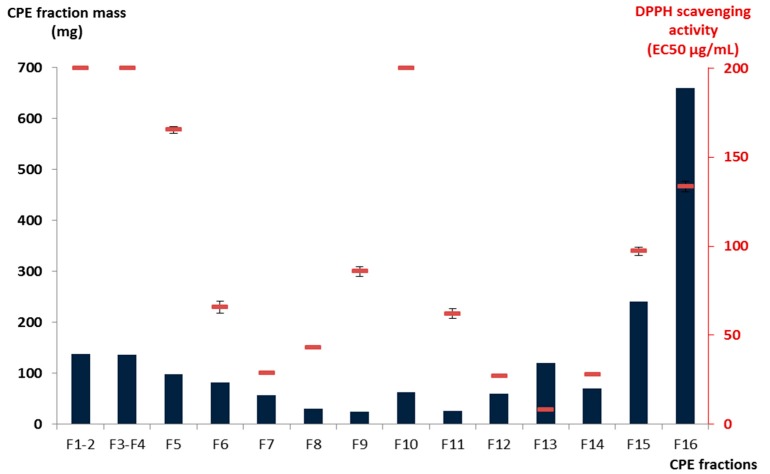
Mass and DPPH radical scavenging activity of the CPE fractions obtained.

The MeOH extract of the aerial parts of *S. varia* exhibited a significant DPPH radical scavenging effect with an EC_50_ value of 92.6 μg/mL. In order to isolate the compounds involved in this antioxidant action, a bioactivity-guided fractionation strategy was applied throughout the separation procedure. Among the five fractions (from A to E) obtained from the initial crude extract by Diaion HP-20 column chromatography, fraction C exhibited the highest DPPH radical scavenging activity with an EC_50_ of 35 ± 2.0 μg/mL (Table 1). This value is close to that obtained for ascorbic acid (EC_50_ 31 ± 0.1 μg/mL). Accordingly, fraction C was further subjected to CPE fractionation and a ^13^C-NMR-based dereplication method was applied to identify the major known compounds responsible for this radical scavenging activity. The biphasic solvent system composed of M*t*BE/MeCN/water (3:3:4, *v*/*v*) in CPE was used in the ascending mode (*i.e.*, the upper organic phase was used as the mobile phase) to recover moderately polar compounds such as flavonoid derivatives from 1.8 g of fraction C. After pooling the collected fractions on the basis of TLC profile similarities, sixteen adjacent sub-fractions (F1–F16) containing simplified mixtures or even pure compounds were obtained. As illustrated in Figure 1, the total mass collected during the CPE elution step from F1 to F15 represented 63.3% of the injected quantity, while 36.7% of the initial fraction C constituents—the most polar ones—were strongly retained in the stationary phase (F16) of the CPE column and obtained by simple emptying of the column. Among these sub-fractions, F13 was the most active against the stable free radical DPPH with an EC_50_ value of 8.0 ± 0.2 μg/mL followed by F7, F12, and F14 exhibiting EC_50_ values of 28.7 ± 0.9, 26.8 ± 0.8, and 27.7 ± 0.5 μg/mL, respectively (Table 1 and Figure 1). The other fractions were significantly less active than the positive reference ascorbic acid.

**Table 1:** DPPH radical scavenging activities of MeOH extract, fractions, and compounds isolated from *S. varia* ^a^.

Fraction	Mass of Fraction		DPPH Radical Scavenging Activity EC_50_ (μg/mL) ^b^
MeOH extract	80 g		92.6 ± 3.2
A	50.5 g		>200
B	4.8 g		50 ± 1.9
C	7.2 g		35 ± 2.0
D	12.4 g		75 ± 2.2
E	4.8 g		>200
**Compounds identified in the fraction (Peak area) ^d^**			
F1–F2	137 mg		>200
F3–F4	136 mg	**1** (85%)	>200
F5	97 mg	**1** (75%), **5** (8%), **6** (11%), **15**, **16**	165.3 ± 1.7
F6	82 mg	**1** (28%), **2**, **4** (16%), **5** (40%), **6** (9%), **15**, **16**	65.7 ± 3.4
F7	57 mg	**5** (62%), **6** (19%), **15**, **16**	28.7 ± 0.9
F8	30 mg	**5** (13%), **6** (34%), **7** (43%)	42.8 ± 0.3
F9	24 mg	**6** (5%), **7** (88.9%)	85.8 ± 2.8
F10	63 mg	**7** (93.7%)	>200
F11	25 mg	**7** (74.5%), **8** (25.5%)	62.0 ± 2.7
F12	60 mg	**7**, **8** (63%)	26.8 ± 0.8
F13	120 mg	**8** (99%)	**8.0 ± 0.2**
F14	70 mg	**8** (73%), **14**, **15**	27.7 ± 0.5
F15	240 mg	**3** (10%), **9** (27%), **11** (10%), **14**	97.2 ± 2.3
F16 (stationary phase)	660 mg	**10** (60%), **11** (5%), **12** (10%), **13** (10%)	133.5 ± 2.8
**DPPH radical scavenging activity EC_50_ (μM) ^b^**			
Compound **5**		43.5 ± 1.5	20.2 ± 0.7
Compound **6**		26.9 ± 1.9	12.5 ± 0.9
Compound **8**		17.8 ± 0.4	8.0 ± 0.2
Compound **11**		37.4 ± 0.8	17.3 ± 0.4
Ascorbic acid ^c^		176.1 ± 0.5	31 ± 0.1

^a^: 50% inhibition not achieved at 200 μg/mL for compounds **1**–**4**, **7**, **9**–**10**, and **12**–**16**; ^b^: Values are presented as the mean ± S.D. (*n* = 3); ^c^: Used as a positive control; ^d^: Peak area in HPLC analytic analysis at 275 nm (when % ≥ 5%).

**Figure 2:**
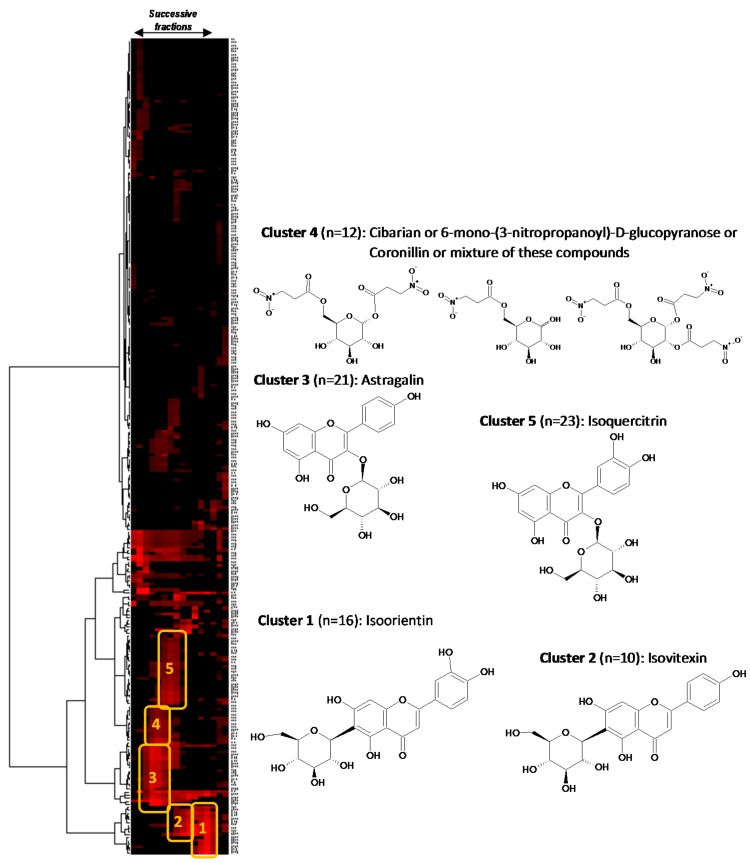
^13^C-NMR chemical shift clusters obtained by applying HCA on CPE fractions.

In parallel to DPPH scavenging assays, fractions F1–F16 were all analyzed by ^13^C-NMR for dereplication [16]. Automatic peak picking and binning of ^13^C signals across spectra resulted in a table with 16 columns (one per fraction) and 267 rows (one per chemical shift bin containing at least one ^13^C signal in at least one fraction). Hierarchical Clustering Analysis (HCA) was applied on the rows of the resulting table. In this way, statistical correlations between ^13^C-NMR resonances belonging to a single structure within the fraction series were easily visualized as “chemical shift clusters” in front of the corresponding dendrograms. As illustrated in Figure 2, several well-defined clusters were intensely colored in red on the resulting two-dimensional HCA correlation map.

Cluster 1 corresponded to an intense cluster of sixteen ^13^C-NMR chemical shifts. After entering these NMR chemical shift values into the database containing the structures and predicted ^13^C-NMR chemical shifts of ≈1200 natural compounds among which 27 known metabolites from *S. varia*, the structure of isoorientin (**8**) was proposed as the first hit of over eight proposals. This structure was successfully confirmed by finding all NMR chemical shifts of isoorientin in raw NMR data of fractions F13 where the intensity of cluster 1 was predominant [17]. Isoorientin was also detected as the major compound in fractions F12 and F14. By applying exactly the same approach, cluster 2 was identified as isovitexin (**7**). Its structure matched as the first hit over 10 proposals from the database and was confirmed by finding all ^13^C chemical shifts in raw NMR data of fractions F9 and F10 [16]. Astragalin (**1**) matched as the first to hit over 25 proposals for cluster 3 [18]. Regarding cluster 4 which corresponded to a cluster of 12 ^13^C-NMR chemical shifts, cibarian, 6-mono-(3-nitropropanoyl)-d-glucopyranose, and coronillin matched as first, second, and third hits, respectively. However, when trying to find the ^13^C-NMR chemical shifts of each of these compounds in the spectra of fractions F4, F5, and F6, the unambiguous assignment of cluster 4 to a molecular structure was not successfully achieved because several of these structurally close nitropropanoyl-d-glucopyranose derivatives were mixed as minor compounds in these fractions already containing predominantly astragalin. Cluster 5 that corresponded to an intense cluster of 21 ^13^C-NMR chemical shifts was successfully assigned to isoquercitrin (**5**) in F7 and F8 [18]. The other NMR chemical shift clusters obtained by HCA did not match any of the compounds stored in the database, hence precluding identification of additional *S. varia* metabolites by dereplication. To conclude on this dereplication work, four major flavonoids of the crude methanolic extract of *S. varia* aerial parts including isoorientin (**8**), isovitexin (**7**), astragalin (**1**), and isoquercitrin (**5**), were rapidly identified by dereplication. Isoorientin was largely predominant in F13 (99% of HPLC purity at 275 nm) and thus was certainly involved in the very low EC_50_ value (8.0 ± 0.2 μg/mL) of this fraction. The significant DPPH radical scavenging activity of isoorientin comparable to that of ascorbic acid was previously reported [19]. Isovitexin (**7**) was the major compound in F9 and F10 (88.9% and 93.7% of HPLC purity at 275 nm, respectively), while astragalin was the major compounds in F4 and F5 (85% and 75% of HPLC purity at 275 nm, respectively) accompanied by a mixture of minor nitropropanoyl-d-glucopyranose derivatives. The EC_50_ values obtained for these fractions, although showing an antioxidant potential, were significantly lower than that of F13 (Figure 1).

**Figure 3:**
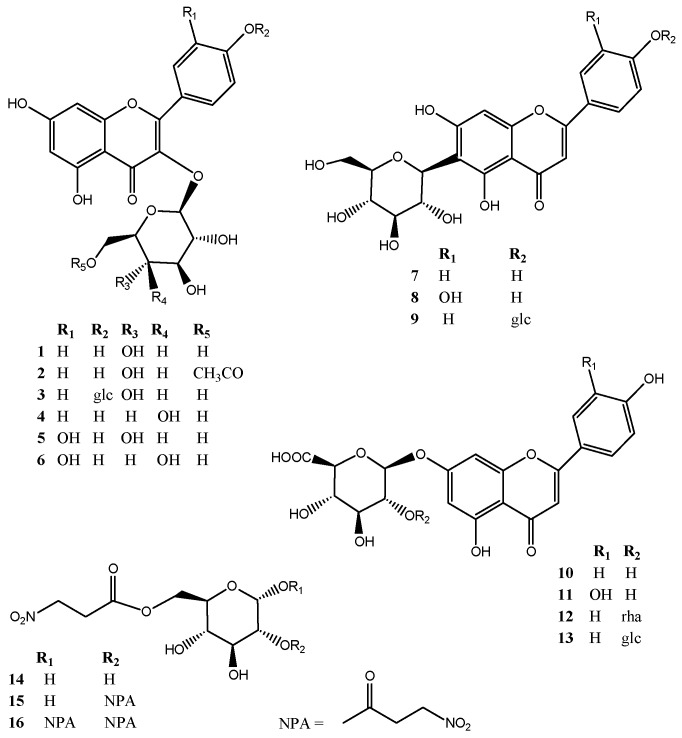
Structures of compounds **1**–**16** isolated from the aerial parts of *Securigera varia*.

In an attempt to better understand if particular molecular structure patterns are directly involved in the DPPH radical scavenging effect of flavonoids, further semi-preparative reversed phase HPLC (RP-HPLC) purification experiments were performed to identify more *S. varia* metabolites in the CPE fraction series. As a result, one new compound (**13**) and twelve known flavonoid glycosides together with three nitropropanoylglucopyranoses were isolated including four flavonoids already identified by dereplication (**1**, **5**, **7**, and **8)**, in addition to kaempferol-3-*O*-(6-*O*-acetyl)-β-d-glucopyranoside (**2**) [20], kaempferol-3,4′-di-*O*-β-d-glucopyranoside (**3**) [21], trifolin (**4**) [22], hyperoside (**6**) [23], isovitexin 4′-*O*-β-d-glucopyranoside (**9**) [7], apigenin 7-*O*-β-d-glucuronopyranoside (**10**) [24], luteolin 7-*O*-β-d-glucuronopyranoside (**11**) [24], 7-*O*-α-l-rhamnopyranosyl-(1→2)-β-d-glucuronopyranoside (**12**) [25], 6-*O*-(3-nitropropanoyl)-β-d-glucopyranoside (**14**), coronillin (**16**) and coronarian (**15**) [10,26] (Figure 3).

All isolated compounds (**1**–**16**) were then evaluated for their DPPH radical scavenging effect. As summarized in Table 1, only compounds **5**, **6**, **8**, and **11** exhibited EC_50_ values ranging from 8.0 to 20.2 μg/mL whereas the 50% DPPH inhibition was not reached even at 200 μg/mL for the other compounds. The purification of fraction F7 that exhibited an EC_50_ value of 28.7 ± 0.9 μg/mL (Table 1 and Figure 1) yielded isoquercitrin (**5**) which was already identified by dereplication, hyperoside (**6**), as well as two minor compounds coronillin (**16**), and coronarian (**15**). The activity of F7 was thus attributed to both **5** and **6** since compounds **15** and **16** were not active at 200 μg/mL (Table 1). The semi-preparative RP-HPLC purification of fraction F6 that showed a moderate radical scavenging activity (EC_50_ 65.7 ± 3.4 μg/mL) afforded compounds **5** and **6** as previously isolated from F7, isovitexin (**7**) as previously identified by dereplication, but also trifolin (**4**) and kaempferol-3-*O*-(6-*O*-acetyl)-β-d-glucopyranoside (**2**). Since compounds **2**, **4**, and **7** were not active at 200 μg/mL, the activity of F6 was also attributed to both **5** and **6** (Table 1). Fraction 8 that showed moderate antioxidant activity with an EC_50_ value of 42.8 ± 0.3 μg/mL also yielded isoquercitrin (**5**), in addition to hyperoside (**6**) and isovitexin (**7**) after semi-preparative RP-HPLC purification, and the activity of this fraction was attributed to hyperoside and isoquercitrin (Table 1). Fraction 10 contained 63 mg of isovitexin (**7**) (93.7% of HPLC purity at 275 nm). Fraction F13 contained 120 mg of isoorientin (**8**) (99% of HPLC purity at 275 nm). The purification of fraction F15 that showed low DPPH radical scavenging activity (EC_50_ 97.2 ± 2.3 μg/mL) led to the isolation of 6-*O*-(3-nitropropanyl)-d-glucopyranoside (**14**), kaempferol-3,4′-di-*O*-β-d-glucopyranoside (**3**), isovitexin 4′-*O*-β-d-glucopyranoside (**9**), in addition to the active compound **8**. The purification of fraction F16 which corresponded to all compounds retained inside the aqueous stationary phase during the CPE fractionation experiment and that exhibited low activity (EC_50_ 133.5 ± 2.8 μg/mL) yielded luteolin 7-*O*-β-d-glucuronopyranoside (**11**), apigenin 7-*O*-α-l-rhamnopyranosyl-(1→2)-β-d-glucuronopyranoside (**12**), apigenin 7-*O*-β-d-glucuronopyranoside (**10**), and compound **13**. Results in Table 1 indicated that compound **11** (EC_50_ 17.3 μg/mL) was the only active compound in fractions F15 and F16. Altogether these results revealed that the radical scavenging activities of the tested flavonoids were correlated with the number and position of phenolic hydroxyl groups in the molecules. Bioactivity data confirmed that *ortho*-dihydroxyl groups in the B-ring and the 2,3-double bond in conjugation with 4-oxo function in the C-ring play a crucial role in radical-scavenging activity in DPPH assay [27,28,29,30,31,32,33,34]. These trends are consistent with less active flavonoids (**1**–**4**, **7**, **9**, **10**, **12**, and **13**) that possess mono-OH substitution in the B-ring. Results indicated that the flavone *C*-glycoside (**8**) is more active than the 7-*O*-flavone glycoside (**11**) that was in agreement with previous findings indicating that glycosylation at C-7 position decreased the radical-scavenging activity [35]. Comparison of antioxidant activity of isoquercitrin (**5**) and hyperoside (**6**) indicated that glycosylation with a galactose unit at C-3 position (**6**) was most favorable for the antioxidant activity than glycosylation with a glucose residue (**5**).

During this study, a new compound (**13**) was isolated as a yellow, amorphous powder. Its HR-ESI-MS spectrum acquired in the positive ion mode showed an [M + Na]^+^ ion at *m*/*z* 631.1283, indicating the molecular formula of C_27_H_28_O_16_. The UV spectrum showed an absorption maximum at 269 and 334 nm, suggesting that **13** is a flavonoid glycoside. The downfield region of the ^1^H-NMR spectrum showed two spin systems exhibiting correlations in the COSY experiment, the first spin system at δ 7.92 (d, 8.7 Hz, H-2′ and H-6′) and 6.96 (d, 8.7 Hz, H-3′and H-5′) and the second one at δ 6.52 (d, 1.8 Hz, H-6) and 6.86 (d, 1.8 Hz, H-8). These two systems together with a singlet at δ 6.69 (H-3) suggested the presence of an apigenin aglycone in compound **13**. Complete assignment of the remaining resonances of the aglycone in the ^13^C-NMR spectrum of **13** was achieved by analysis of the HSQC and HMBC spectral data, which confirmed the presence of apigenin (4′,5,7-trihydroxyflavone) [25]. A full list of the corresponding assignments is given in the experimental section.

The sugar region of the ^1^H-NMR spectrum showed the presence of two sugar units, with anomeric proton signals at δ 5.44 (d, 7.0 Hz, H-1′′) and δ 4.70 (d, 7.8 Hz, H-1′′′) correlating in the HSQC experiment with the anomeric carbons at δ 98.3 and 103.6. Analysis of 2D NMR experiments revealed the presence of a terminal β-d-glucopyranosyl unit (δ_H-1′′_ 5.44) and a β-d-glucuronic acid in pyrane form (δ_H-1′′′_ 4.70). Complete assignment of each glycoside proton system was achieved by analysis of COSY, HSQC, and HMBC experiments. Their β-configuration was evident from the large coupling constant of the anomeric protons. The pronounced downfield shift of C-2′′ of the glucuronic acid (δ 82.7) and the HMBC correlation between H-1′′′ and C-2′′ indicated that the glucopyranose unit was linked to C-2′′ of the glucuronopyranose unit. A correlation between H-1′′ of glcA and δ_C_ 162.9 in the HMBC spectrum of **13** defined C-7 of apigenin as the site of *O*-glycosylation. Therefore, the structure of compound **13** was determined as apigenin 7-*O*-β-d-glucopyranosyl-(1→2)-β-d-glucuronopyranoside.

## 3. Experimental Section

### 3.1. Solvents and Reagents

1,1-diphenyl-2-picrylhydrazyl (DPPH), ascorbic acid, deuterated dimethyl sulfoxide (DMSO-*d*_6_), and deuterated methanol (methanol-*d*_4_) were purchased from Sigma-Aldrich (Saint-Quentin, France). Methyl *tert*-butyl ether (M*t*BE), acetonitrile (MeCN), methanol (MeOH), trifluoroacetic acid (TFA), chloroform (CHCl_3_), and dimethyl sulfoxide (DMSO) of analytical quality were purchased from VWR (Fontenay-sous-Bois, France). Deionized water was used to prepare all aqueous solutions.

### 3.2. Plant Material and Extraction

The aerial parts of *Securigera varia* (L.) Lassen were collected in Reims (Moulin de la Housse), north-east of France in April 2013, and dried at room temperature. A flowering voucher specimen is deposited in the Herbarium of the Botanic laboratory-Faculty of Pharmacy, University of Reims Champagne-Ardenne, under the sheet reference MA-2014-01, the identity of which was verified by Dr A. Alabdul Magid.

The dried and powdered aerial parts of *S. varia* (500 g) were macerated in methanol (15 L) at room temperature for 48 h. After filtration, the organic phase was evaporated to dryness under reduced pressure to afford 80 g of MeOH extract. This starting crude extract was evaluated for its radical scavenging activity.

### 3.3. Fractionation Experiments

**Figure 4:**
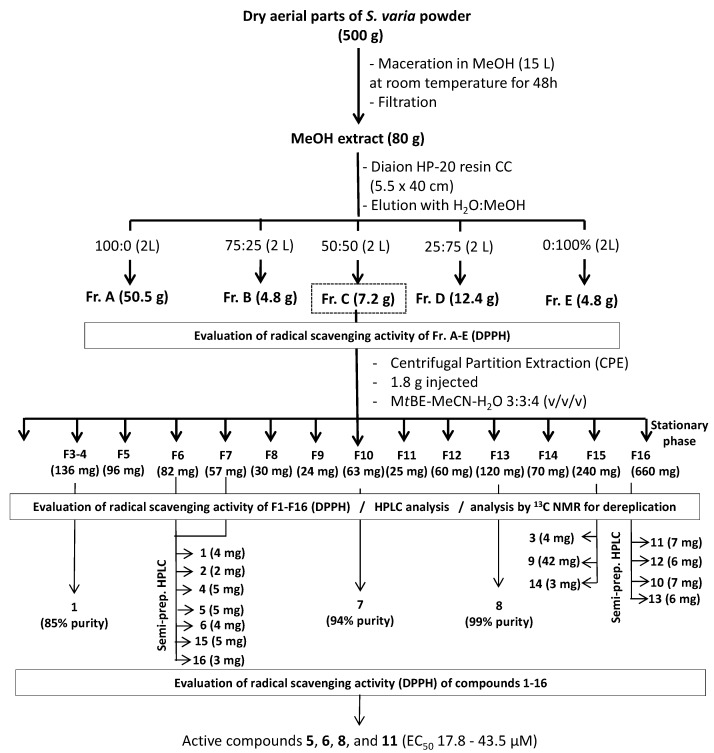
Extraction scheme for isolation of antioxidant compounds from *S. varia* aerial parts.

The crude MeOH extract of *S. varia* (80 g) was dissolved in 500 mL of distilled water and rapidly fractionated by Column Chromatography (CC) on a Diaion HP-20 resin (5.5 × 40 cm, Sigma-Aldrich, Saint-Quentin, France) eluted successively with 2 L of 0%, 25%, 50%, 75%, and 100% MeOH in H_2_O, to give five fractions (A: 50.5 g, B: 4.8 g, C, 7.2 g, D: 12.4 g, and E: 4.8 g, respectively) (Figure 4). All fractions were also tested for their radical scavenging activity, and the most active (fraction C) was further fractionated by Centrifugal Partition Extraction (CPE) on a lab-scale column of 303 mL capacity (FCPE300^®^, Rousselet Robatel Kromaton, Annonay, France) containing seven circular partition disks, engraved with a total of 231 oval partition twin-cells (≈1 mL per twin cell). The biphasic solvent system (3 L) was prepared by mixing M*t*BE, MeCN and water in the proportion 3:3:4 (*v*/*v*/*v*) in a separatory funnel. The CPE column was filled with the aqueous stationary phase at 200 rpm by using a KNAUER Preparative 1800 V7115 pump (Berlin, Germany). After accelerating the rotation to 1200 rpm, 1.8 g of fraction C dissolved in 25 mL of a mixture of the aqueous and organic phases (2:3; *v*/*v*) were injected through a 30 mL sample loop. The organic mobile phase then was pumped at 20 mL/min in the ascending mode. Fractions of 20 mL were collected over the whole experiment by using a Pharmacia Superfrac collector (Uppsala, Sweden) and were combined according to their thin layer chromatography (TLC) profiles. TLC was performed on pre-coated silica-gel 60F_254_ Merck with the migration solvent system CHCl_3_/MeOH/H_2_O (8:2:0.2; *v*/*v*/*v*), and visualized under UV light at 254 and 365 nm and by spraying the dried plates with 50% H_2_SO_4_, followed by heating. As a result, 16 sub-fractions (F1–F16) were obtained.

### 3.4. ^13^C-NMR-Based Dereplication of the CPE-Generated Fractions

A recently developed dereplication method was used to rapidly identify the major metabolites present in the fractions obtained from the CPE experiment [15]. Briefly, an aliquot of each fraction (≈20 mg) was dissolved in 600 μL methanol-*d*_4_ and analyzed by ^13^C-NMR at 298 K on a Bruker Avance AVIII-600 spectrometer (Karlsruhe, Germany) equipped with a TXI cryoprobe. ^13^C-NMR spectra were acquired at 150.91 MHz. A standard zgpg pulse sequence was used with an acquisition time of 0.909 s and a relaxation delay of 3 s. For each sample, 1024 scans were co-added to obtain a satisfactory signal-to-noise ratio. The spectral width was 238.9070 ppm and the receiver gain was set to the highest possible value. A 1 Hz line broadening filter was applied to each FID prior to Fourier transformation. Then spectra were manually phased and baseline corrected using the TOPSPIN 3.2 software (Bruker) and calibrated on the central resonance of MeOH-*d*_4_ (δ 47.60 ppm). The absolute intensities of all ^13^C-NMR signals were automatically collected and each peak list was stored as a text file. The collected peaks in the fraction series were subsequently binned by using a locally developed computer script written in Python language. Its principle was to divide the ^13^C spectral width (from 0 to 220 ppm) into regular chemical shift intervals (∆δ = 0.2 ppm), and to associate the absolute intensity of each ^13^C peak to the corresponding bin. The resulting table was imported into the PermutMatrix version 1.9.3 software (LIRMM, Montpellier, France) for clustering analysis on raw peak intensity values. The classification was performed on the rows only, *i.e.*, on the chemical shift bins. The Euclidian distance was used to measure the proximity between samples and the Ward’s method was applied to agglomerate the data. The resulting ^13^C chemical shift clusters were visualized as dendrograms on a two-dimensional map. The higher the intensity of ^13^C-NMR peaks, the brighter the color on the map (Figure 2). In parallel, a literature survey was performed to obtain names and structures for a maximum of *S. varia* metabolites already described in the literature. In total, 27 compounds were found, mostly including flavonoids and to a lesser extent nitropropanoylglucopyranoses and cardenolides. They were added to a locally built ^13^C-NMR chemical shift database (ACD/NMR Workbook Suite 2012 software, ACD/Labs, ON, Canada) comprising the structures and predicted chemical shifts of other natural products (*n* = 1200 in June 2015). For metabolite identification, each ^13^C chemical shift cluster obtained from HCA was manually submitted to the structure search engine of the database management software. A ^13^C-NMR chemical shift tolerance of ±2 ppm was used, and the computation time for each search was less than 1 s.

### 3.5. Isolation and Structure Determination of Minor Compounds

Semi-preparative HPLC was performed on a Dionex system equipped with an ASI-100 autosampler, a P580 pump, a Phenomenex column (Luna 5 μm C18 100 Å, 250 mm × 15 mm) a diode array detector UVD 340S and the Chromeleon software. The mobile phases consisted of 0.025% TFA in H_2_O and MeCN. A flow rate of 5 mL/min was used and the chromatograms were monitored at 205, 225, 275, and 350 nm. Fractions F6 and F7 obtained by CPE were further purified by using the following gradient: 20% MeCN increased to 30% in 25 min to yield compounds **16** (*R*t 11.3 min; 3 mg), **15** (*R*t 12.3 min; 5 mg), **1** (*R*t 13.1 min; 4 mg), **5** (*R*t 14.3 min; 5 mg), **6** (*R*t 16.3 min; 4 mg), **4** (*R*t 17.5 min; 5 mg) and **2** (*R*t 20.5 min; 2 mg). The purification of fraction F15 by semi-preparative HPLC using the gradient: MeCN increased from 5% to 15% in 12 min then to 25% in 18 min led to the isolation of compounds **14** (*R*t 11.5 min; 3 mg), **3** (*R*t 12.5 min; 4 mg), and **9** (*R*t 20.5 min; 42 mg). The purification of the aqueous phase (F16) by semi-preparative HPLC using the gradient: MeCN increased from 15% to 25% in 25 min yielded compounds **11** (*R*t 18.7 min; 7 mg), **12** (*R*t 20.5 min; 6 mg), **10** (*R*t 23.9 min; 7 mg), and **13** (*R*t 17.2 min; 6 mg).

The molecular structures of the purified compounds were elucidated by 1D ^1^H and ^13^C and 2D COSY, HSQC, and HMBC NMR analyses in MeOH-*d*_4_ or DMSO-*d*_6_ on a Bruker Avance DRX III-500 by using standard Bruker pulse sequences. ESI-MS experiments were performed using a Micromass Q-TOF micro instrument (Manchester, UK). Analytical HPLC was performed to determine the HPLC-purity (at λ = 210 and 275 nm) of the isolated compounds on the same Dionex system. The chromatographic column was a Luna (5 μm C18 100 Å, 250 mm × 4.6 mm) and the elution was performed by using the following gradient: 10% MeCN increased to 30% in 25 min at 1 mL/min. Optical rotations were determined in MeOH with a Perkin-Elmer 341 polarimeter. UV spectra were recorded on Shimadzu UV-2450 spectrophotometer in MeOH. All sugar moieties were unambiguously identified by using an acid hydrolysis method as previously described [36]. Briefly, 500 mg of fraction C was refluxed with 50 mL of 2M TFA for 3 h. After extraction with ethyl acetate (3 × 15 mL), the aqueous layer was evaporated to furnish the monosaccharide residue (160 mg). Four sugars were identified as d-glucuronic acid, d-glucose, d-galactose, and l-rhamnose by comparison with authentic samples on TLC and by measurement of the optical rotation of each purified sugar and comparison with references.

### 3.6. Free Radical Scavenging Activity

The radical scavenging activity of the initial crude extract, fractions, and purified compounds was measured using the DPPH method [37]. Briefly, 5 μL of different concentrations of the samples (dissolved in DMSO) were added to 95 μL of a DPPH solution (158 μM, dissolved in EtOH 50%). The reaction proceeded for 30 min at 37 °C on a 96-well microplate. The absorbance was then read at λ 515 nm on a Fluostar omega microplate reader (BMG labtech, Ortenberg, Germany). The DPPH inhibition percentage was calculated as followed: % inhibition ((Ab_control_ − Ab_sample_)/Ab_control_) × 100. A DPPH solution in EtOH 50% was used as a control. The curve of the % scavenging activity against the concentration of sample was prepared by MSExcel based program to obtain the EC_50_ (concentration required to obtain a 50% antioxidant effect). Samples were prepared at concentrations of 100, 50, 25, 6.2 and 3.1 μg/mL. All tests were conducted in triplicate. Experimental data were expressed as mean ± standard deviation (*n* = 3 replicates). Ascorbic acid was used as a positive control.

### 3.7. Identification Data of Compound **13**

Yellow, amorphous powder, ${\left[\alpha \right]}_{D}^{20}$ +25.5 (*c* 0.1, MeOH); UV (MeOH) λ_max_ nm: 268 (ε = 2245 mol^−1^·L·cm^−1^), 332 (ε = 2555 mol^−1^·L·cm^−1^); IR ν_max_ (film) 3359, 2946, 2834, 1660, 1454, 1116, 1029 cm^−1^; ^1^H-NMR (CD_3_OD, 500 MHz): δ 6.69 (1H, s, H-3), 6.52 (1H, d, *J* = 1.8 Hz, H-6), 6.86 (1H, d, *J* = 1.8 Hz, H-8), 7.92 (2H, d, *J* = 8.7 Hz, H-2′, H-6′), 6.96 (2H, d, *J* = 8.7 Hz, H-3′, H-5′), 5.44 (1H, d, *J* = 7.0 Hz, H-1′′), 3.83 (1H, t, *J* = 7.2 Hz, H-2′′), 3.77 (1H, t, *J* = 8.8 Hz, H-3′′), 3.76 (1H, t, *J* = 8.8 Hz, H-4′′), 4.15 (1H, d, *J* = 8.8 Hz, H-5′′), 4.70 (1H, d, *J* = 7.8 Hz, H-1′′′), 3.27 (1H, t, *J* = 8.6 Hz, H-2′′′), 3.43 (1H, t, *J* = 8.6 Hz, H-3′′′), 3.40 (1H, t, *J* = 8.6 Hz, H-4′′′), 3.31–3.33 (1H, m, H-5′′′), 3.57 (1H, dd, *J* = 11.9–2.1 Hz, H-6′′′), 3.66 (1H, dd, *J* = 11.9–5.2 Hz, H-6′′′); ^13^C-NMR (DMSO-*d*_6_, 125 MHz): δ 164.7 (C-2), 105.0 (C-3), 182.4 (C-4), 161.3 (C-5), 99.9 (C-6), 162.9 (C-7), 95.5 (C-8), 157.3 (C-9), 105.9 (C-10), 121.5 (C-1′), 129.0 (C-2′, C-6′), 116.4 (C-3′, C-5′), 161.9 (C-4′), 98.3 (C-1′′), 82.7 (C-2′′), 75.4 (C-3′′), 71.3 (C-4′′), 75.2 (C-5′′), 170.6 (C-6′′), 103.6 (C-1′′′), 75.0 (C-2′′′), 76.5 (C-3′′′), 69.9 (C-4′′′), 77.3 (C-5′′′), 60.9 (C-6′′′); HR-ESI-MS *m*/*z*: 631.1283 [M + Na]^+^ (calcd for C_27_H_28_O_16_Na, 631.1275).

## 4. Conclusions

In this study, CPE separation combined to ^13^C-NMR-based dereplication and DPPH radical scavenging detection was applied to fractionate a methanolic extract from *S. varia* aerial parts into fractions containing components with various polarities. The dereplication work applied on CPE fractions allowed the fast identification of four major flavonoids including isoorientin (**8**), isovitexin (**7**), astragalin (**1**), and isoquercitrin (**5**). Purification of the active fractions by semi-preparative RP-HPLC afforded one new compound (**13**) and twelve known flavonoid glycosides together with three nitropropanoylglucopyranoses. Four flavonoids were identified as the main antioxidant compounds (**5**, **6**, **8**, and **11**), in particular compound **8** which showed a higher scavenging effect than the positive reference ascorbic acid. It should also be noted that 136 mg astragalin, 63 mg isovitexin, and 120 mg isoorientin were obtained as pure compounds from 1.8 g crude extract in a one-step CPE elution experiment in less than 1 h and quickly identified by ^13^C-NMR-based dereplication. The method is simple, fast, and without complex solvent system or gradient elution.
